# Distinct role of AtCuAOβ- and RBOHD-driven H_2_O_2_ production in wound-induced local and systemic leaf-to-leaf and root-to-leaf stomatal closure

**DOI:** 10.3389/fpls.2023.1154431

**Published:** 2023-04-21

**Authors:** Ilaria Fraudentali, Chiara Pedalino, Riccardo D’Incà, Paraskevi Tavladoraki, Riccardo Angelini, Alessandra Cona

**Affiliations:** ^1^ Department of Science, University Roma Tre, Rome, Italy; ^2^ Istituto Nazionale Biostrutture e Biosistemi (INBB), Rome, Italy; ^3^ NBFC, National Biodiversity Future Center, Palermo, Italy

**Keywords:** CuAO, RBOH, polyamines, ROS, hydrogen peroxide, wounding, stomatal closure, systemic stress responses

## Abstract

Polyamines (PAs) are ubiquitous low-molecular-weight aliphatic compounds present in all living organisms and essential for cell growth and differentiation. The developmentally regulated and stress-induced copper amine oxidases (CuAOs) oxidize PAs to aminoaldehydes producing hydrogen peroxide (H_2_O_2_) and ammonia. The *Arabidopsis thaliana* CuAOβ (AtCuAOβ) was previously reported to be involved in stomatal closure and early root protoxylem differentiation induced by the wound-signal MeJA *via* apoplastic H_2_O_2_ production, suggesting a role of this enzyme in water balance, by modulating xylem-dependent water supply and stomata-dependent water loss under stress conditions. Furthermore, AtCuAOβ has been shown to mediate early differentiation of root protoxylem induced by leaf wounding, which suggests a whole-plant systemic coordination of water supply and loss through stress-induced stomatal responses and root protoxylem phenotypic plasticity. Among apoplastic ROS generators, the D isoform of the respiratory burst oxidase homolog (RBOH) has been shown to be involved in stress-mediated modulation of stomatal closure as well. In the present study, the specific role of AtCuAOβ and RBOHD in local and systemic perception of leaf and root wounding that triggers stomatal closure was investigated at both injury and distal sites exploiting *Atcuaoβ* and *rbohd* insertional mutants. Data evidenced that AtCuAOβ-driven H_2_O_2_ production mediates both local and systemic leaf-to-leaf and root-to-leaf responses in relation to stomatal movement, *Atcuaoβ* mutants being completely unresponsive to leaf or root wounding. Instead, RBOHD-driven ROS production contributes only to systemic leaf-to-leaf and root-to-leaf stomatal closure, with *rbohd* mutants showing partial unresponsiveness in distal, but not local, responses. Overall, data herein reported allow us to hypothesize that RBOHD may act downstream of and cooperate with AtCuAOβ in inducing the oxidative burst that leads to systemic wound-triggered stomatal closure.

## 1 Introduction

A complex integration of different environmental and endogenous signals coordinates stomatal responses, ensuring a balance between CO_2_ uptake and water loss. Stress-driven modulation of stomata movement is a primary response to achieve rapid plant acclimation to abiotic and biotic stress conditions, in order to regulate transpiration rate and restrict microbial entry into leaves. Abscisic acid (ABA; Bharath et al., 2021), salicylic acid (SA; Khokon et al., 2011), and jasmonic acid (JA; Fraudentali et al., 2021a) are key players in stress-mediated stomatal closure through signaling pathways involving biologically active compounds that integrate stimuli and coordinate each other to achieve proper stomatal responses during stress combination (Iqbal et al., 2021; Rivero et al., 2022).

In this context, upon stress perception, ABA and JA trigger the production of reactive oxygen species (ROS) (Devireddy et al., 2021), which are key players in controlling stomatal movement (Song et al., 2014). One of the earliest events of stomatal closure is ROS accumulation in the apoplast (Qi et al., 2018), which activates ROS-dependent Ca^2+^ channels, thus increasing cytosolic Ca^2+^ levels and triggering the signal transduction cascade, which leads to the closure of stomatal pore (Pei et al., 2000).

Nonetheless, the perception of ROS and the immediate downstream elements of their signaling are mostly unknown. Recently, at least one H_2_O_2_ receptor involved in stomatal and ROS wave regulation was identified in Arabidopsis, which is H_2_O_2_-induced Ca^2+^ increases 1 (HPCA1; Fichman et al., 2022). Moreover, what is known is that ROS can act locally or spread to distal sites far from the place of biosynthesis as a wave (Mittler et al., 2011). Among ROS, hydrogen peroxide (H_2_O_2_) has the longest half-life and, for this reason, can signal among cells in the apoplast as a long-distance communication molecule (Benkő et al., 2022).

Respiratory burst oxidase homologs NADPH oxidases (RBOHs) and amine oxidases (AOs) greatly contribute, along with other enzymatic systems such as peroxidases and oxalate oxidases (Cona et al., 2006), to H_2_O_2_ accumulation, either intracellularly or extracellularly. RBOHs are plasma membrane proteins that catalyze the formation of superoxide anion ( $O_{2}.-$ ), which converts, spontaneously or thanks to the superoxide dismutase activity, into H_2_O_2_ (Choudhury et al., 2017), which accumulates in the apoplast (Suzuki et al., 2011). Among Arabidopsis RBOH proteins, isoforms RBOHF and RBOHD play crucial roles in both biotic and abiotic stress responses (Farvardin et al., 2020). However, it has been reported that RBOHF is constitutively expressed in the vascular tissue and hydathodes and downregulated by ABA in guard cells (Kwak et al., 2003; Morales et al., 2016). On the contrary, ABA-induced (Kwak et al., 2003) isoform RBOHD is highly constitutively expressed in different leaf and root areas, as well as in guard cells (Morales et al., 2016) where it mediates both local and systemic responses to light stress (Devireddy et al., 2018). Furthermore, it has been reported that RBOHD is involved in systemic, but not local, stomatal responses to wounding (Devireddy et al., 2020).

AOs catalyze the oxidative de-amination of polyamines (PAs), which are ubiquitous aliphatic polycations involved in key events of cell life. AOs include two distinct enzyme classes, copper amine oxidases (CuAOs) and flavin adenine dinucleotide (FAD)-dependent polyamine oxidases (PAOs). H_2_O_2_ is a shared by-product in all AO-catalyzed reactions, which contribute to different developmental processes and stress-induced responses (Ghuge et al., 2015a). Indeed, H_2_O_2_ deriving from intra- and extracellular AOs has been reported to be involved in the control of stomatal movement in several plant species (Tavladoraki et al., 2016; Fraudentali et al., 2021a). Among Arabidopsis CuAOs (AtCuAOs), the vacuolar AtCuAOδ and the peroxisomal AtCuAOζ have been reported to mediate ABA-induced stomatal closure (Qu et al., 2014; Fraudentali et al., 2019), while the apoplastic AtCuAOβ has been involved in MeJA-induced stomatal closure (Fraudentali et al., 2021b). Notably, the constitutive and MeJA-inducible expression of AtCuAOβ in guard cells and in protoxylem vessels, along with its involvement in MeJA/wound-triggered early root protoxylem differentiation (Ghuge et al., 2015b; Fraudentali et al., 2020a), suggests a role of this enzyme in water balance homeostasis, by modulating xylem-dependent water supply and stomata-dependent water loss under stress conditions.

Several studies have proposed a correlated action of AO and RBOH enzymes in different plant responses (Benkő et al., 2022) through mechanisms in which AO- and RBOH-dependent signaling pathways may be interconnected and may have an impact on each other. Particularly, it has been hypothesized that apoplastic PAO activity modulates RBOH-mediated ROS production in a positive feedback loop, which leads to the amplification of apoplastic ROS accumulation (Gémes et al., 2016). In this context, given the shared role of RBOHD and AtCuAOβ as apoplastic ROS sources, it is of great interest to evaluate the possible interplay of these two proteins in wound-triggered stomatal movement modulation.

In this study, the distinct role of AtCuAOβ and RBOHD in local and systemic wound-induced stomatal closure has been investigated. To this purpose, we mechanically wounded cotyledonary-leaves or roots of Arabidopsis *Atcuaoβ* and *rbohd* loss-of-function mutant seedlings and analyzed stomatal modulation separately in wounded cotyledonary-leaf (local responses) and in unwounded cotyledonary-leaves after leaf or root injury (distal responses).

## 2 Materials and methods

### 2.1 Plant materials, growth conditions, and treatments

*Arabidopsis thaliana* ecotype Columbia-0 (Col-0) was utilized as the wild type (WT). Two Arabidopsis Col-0 T-DNA insertion lines of the here analyzed *CuAO* gene At4g14940 (*AtCuAOβ*, TAIR accession no. 2129519), specifically *Atcuaoβ.1* (SALK_145639.55.25.x; TAIR accession number 1005841762) and *Atcuaoβ.3* (SALK_082394.32.30.x, TAIR accession number 1005822711), were acquired from the SALK Institute Genomic Analysis Laboratory (http://signal.salk.edu/tabout.html accessed on 15 September 2022) and previously characterized [*Atcuaoβ.1* (Ghuge et al., 2015b); *Atcuaoβ.3* (Fraudentali et al., 2021b)]. The Arabidopsis Col-0 T-DNA insertion line of the here analyzed *RBOHD* gene At5g47910 (*RBOHD*, TAIR accession no. 2160916), specifically *rbohd*, was gently provided by Giulia De Lorenzo, University of Rome “La Sapienza” (Torres et al., 2002; Galletti et al., 2008). *AtCuAOβ-promoter::GFP-GUS* transgenic plants here analyzed were also previously described (Ghuge et al., 2015b; Ghuge et al., 2015c).

Plants were cultivated in a growth chamber set at 23°C under long-day conditions (16/8 h photoperiod; 150 µmol m^−2^ s^−1^ and 55% relative humidity). For *in vitro* growth, seeds were sterilized at the surface as previously reported (Valvekens et al., 1988; Ghuge et al., 2015b; Fraudentali et al., 2019; Fraudentali et al., 2020b). Seeds were then stratified in the dark at 4°C for 2 days before being sowed in 1/2 Murashige and Skoog (MS) salt mixture (pH 5.7) supplemented with 0.5 (*w/v*) sucrose, 0.8% (*w/v*) agar (solid medium), and 50 µg/ml kanamycin (only when antibiotic selection was needed).

Seven-day-old seedlings grown *in vitro* on solid medium supplemented with kanamycin were used to perform the histochemical GUS analysis as reported in detail hereafter. For the analysis of inducible tissue-specific gene expression 5 min or 30 min after leaf or root wounding, *AtCuAOβ-promoter::GFP-GUS* 6-day-old seedlings were immersed for 1 day in liquid medium contained inside multiwell plates. Subsequently, the leaf or the root of each seedling was injured using pointed scissors or tweezers with flattened ends, respectively, and 5 min or 30 min were awaited. Samples were analyzed under a light microscope (LM).

*RBOHA* (AT5G07390) and *RBOHF* (AT1G64060) RT-quantitative PCR (RT-qPCR) analyses were performed in 7-day-old WT and *rbohd* seedlings grown *in vitro* on solid medium. Plant samples for constitutive gene expression studies were harvested, frozen in liquid nitrogen, and kept at −80°C until RNA extraction.

Measurements of stomatal aperture levels were executed in 7-day-old WT, *Atcuaoβ.1*, *Atcuaoβ.3*, and *rbohd* seedlings grown on solid medium and examined under control conditions and after leaf or root wounding with or without *N,N^1^*-dimethylthiourea (DMTU; 100 µM) or diphenyleneiodonium (DPI; 50 µM), as reported in more detail further below.

ROS detection in guard cells was performed in 7-day-old WT, *Atcuaoβ.1*, *Atcuaoβ.3*, and *rbohd* seedlings grown on solid medium and examined under control conditions and after leaf or root wounding with or without 100 µM DMTU, as reported in more detail further below.

### 2.2 Histochemical analysis of GUS assay

GUS staining was executed as previously reported (Jefferson, 1987) with slight modifications (Fraudentali et al., 2020b). In detail, 5 min and 30 min after leaf or root wounding, samples were immersed in 90% (*v/v*) ice-cold acetone for pre-fixation for 30 min, washed out three times with sodium phosphate buffer (50 mM, pH 7.0) and then soaked in staining solution [1 mM 5-bromo-4-chloro-3-indolyl-β-D-glucuronide, 2.5 mM potassium ferrocyanide, 2.5 mM potassium ferricyanide, 0.1% (*v/v*) Triton X-100, and 10 mM EDTA in sodium phosphate buffer (50 mM, pH 7.0)]. GUS staining proceeded until a difference was observed in staining intensity between wounded and unwounded seedlings under the microscope (40 min). Chlorophyll extraction was carried out by rinsing off samples with ethanol/acetic acid in a ratio of 1:3 (*v/v*) for 30 min, then with ethanol/acetic acid in a ratio of 1:1 (*v/v*) for 30 min, and lastly with 70% ethanol for another 30 min. Samples were kept in 70% ethanol at 4°C, before being analyzed under LM. Images were taken with a Zeiss Axiophot 2 microscope equipped with a Leica DFC450C digital camera. For quantifying changes in GUS activity in guard cells, acquired images were analyzed by selecting regions of interest (ROI) employing ImageJ software (National Institutes of Health, Bethesda, Maryland, USA) and measuring mean gray values, plotted as the percentage of the ratio between treated and control samples. Data were calculated as means ± SD of mean gray values.

### 2.3 Measurement of stomatal aperture levels

Stomatal aperture level measurements were carried out as previously reported (Shang et al., 2016) with modifications (Fraudentali et al., 2021b), as described in detail hereafter. In order to allow stomatal opening, 7-day-old WT, *Atcuaoβ.1*, *Atcuaoβ.3*, and *rbohd* seedlings grown *in vitro* on solid medium were immersed in opening solution [30 mM KCl and 10 mM MES-Tris (pH 6.15)] for 3 h and left in the growth chamber under light conditions. Successively, the opening solution was removed and samples were soaked in liquid medium, with or without treatment performed as follows: leaf wounding; root wounding; 100 µM DMTU; leaf wounding + 100 µM DMTU; root wounding + 100 µM DMTU; 50 µM DPI; leaf wounding + 50 µM DPI; root wounding + 50 µM DPI. Regarding leaf wounding, stomatal aperture variations in the wounded cotyledonary-leaf were considered as local response, while stomatal aperture variations in the unwounded cotyledonary-leaf were considered as distal response. For time course analysis, seedlings were incubated for 5 min, 15 min, 30 min, 1 h, 3 h, and 24 h in the growth chamber. After each time point, seedlings were incubated in a fixing solution (1% glutaraldehyde, 10 mM NaPi, pH 7.0, 5 mM MgCl_2_, and 5 mM EDTA) for 30 min under light conditions. Images of stomatal pore outlines in the focal plane were taken with a Zeiss Axiophot 2 microscope equipped with a Leica DFC450C digital camera at the magnification of 20×. A digital ruler (ImageJ software) was used to measure width and length of stomatal pores, as their aperture levels were expressed as width/length ratio.

### 2.4 *In situ* detection of reactive oxygen species in guard cells

A chloromethyl derivative of 2’,7’-dichlorodihydrofluorescein diacetate (CM-H_2_DCFDA; Molecular Probes, Invitrogen) was used to analyze ROS production in guard cells as previously reported (Fraudentali et al., 2021b). Seven-day-old WT, *Atcuaoβ.1*, *Atcuaoβ.3*, and *rbohd* seedlings grown *in vitro* on solid medium were incubated in multiwell plates for 3 h in the assay solution [5 mM KCl, 50 µM CaCl_2_, and 10 mM MES-Tris (pH 6.15)] and kept under light in the growth chamber. Successively, 10 µM CM-H_2_DCFDA was added to the assay solution and seedlings were incubated for 30 min at room temperature. After this period, the excess dye was rinsed off twice using fresh assay solution and then seedlings were soaked in liquid medium with or without treatment performed as follows: leaf wounding; root wounding; 100 µM DMTU; leaf wounding + 100 µM DMTU; root wounding + 100 µM DMTU. Seedlings were incubated under light for 1 h. Images were taken with Laser Scanning Confocal Microscopy (LSCM), using a Leica TCS-SP5 equipped with an Argon laser [Excitation: 492–495 nm; Emission: 517–527 nm (CM-H_2_DCFDA), 650–700 nm (chlorophyll auto-fluorescence)] and the software Leica Application Suite Advanced Fluorescence (LAS-AF; Leica Microsystems, Wetzlar, Germany). Shown images are composed of bright field, CM-H_2_DCFDA, and chlorophyll emission channel merged together. To measure the relative fluorescence intensity of guard cells, acquired images were analyzed by selecting ROI and counting pixel numbers in the green channel employing ImageJ software. Data were calculated as means ± SD of pixel intensities.

### 2.5 RNA extraction, RT-PCR, and RT-quantitative PCR

Total RNA was isolated from WT and *rbohd* whole seedlings (100 mg) using GENEzol^®^ Reagent (Invitrogen). Afterward, RNA samples were treated with RNase-Free DNase Set (QIAGEN) to eliminate traces of genomic DNA. cDNA synthesis and PCR amplification were carried out using *GoTaq^®^ 2-Step RT-qPCR System200* (Promega). Quantitative expression profiles of *RBOHA* and *RBOHF* were determined by RT-quantitative PCR (RT-qPCR) using a Corbett Rotor-Gene 6000 (Corbett Life Science, QIAGEN) setting up the following program: 95°C for 2 min then 40 cycles of 95°C for 7 s and 60°C for 40 s. The melting program ramps from 60°C to 95°C, rising by 1°C each step. Ubiquitin-conjugating enzyme 21 (UBC21, At5g25760) was used as the reference gene. Primer sequences used for *RBOHA*, *RBOHF*, and *UBC21* are specified in **Table 1**. Data analysis was performed by the Corbett Rotor-Gene 6000 Application Software (version 1.7, Build 87; Corbett Life Science, QIAGEN). Fold change in the expression of *RBOHA* and *RBOHF* genes was calculated according to the ΔΔCq method as previously described (Livak and Schmittgen, 2001; Fraudentali et al., 2019).

**Table 1:** Primers used for RT-qPCR analysis on whole WT and *rbohd* seedlings.

1. Name of primer	2. Sequence of primer
***UBC21-for***	5′-CTGCGACTCAGGGAATCTTCTAA-3′
***UBC21-rev***	5′-TTGTGCCATTGAATTGAACCC-3′
***RBOHA-qPCR-for***	5′-CATTTCGCTAGGCCAAACTG-3′
***RBOHA-qPCR-rev***	5′-TTCACTAACCCAGCTGCTCCA-3′
***RBOHF-qPCR-for***	5′-GGTGTCATGAACGAAGTTGCA-3′
***RBOHF-qPCR-rev***	5′-AATGAGAGCAGAACGAGCATCA-3′

### 2.6 Statistics

A minimum of 15 plants from three independent experiments were used for GUS staining analysis of tissue-specific gene expression. Images shown were chosen from a single representative experiment. For measurements of stomatal aperture levels, three independent experiments were carried out for each treatment on all analyzed genotypes. For each treatment and time point, five leaves of similar size were harvested from different seedlings of each genotype. In this series of experiments, each of the five leaves derived from all three independent experiments was treated as a biological replicate, making a total of 15 biological replicates for each genotype and treatment (*n* = 15). Four randomly chosen fields (430 μm × 325 μm) for each leaf were taken and at least 60 stomatal pores were measured. The mean values were used in the statistical analysis. CM-H_2_DCFDA staining analysis was performed using LSCM on seedlings from three independent experiments. For each experiment, five leaves of similar size were harvested from different seedlings for each genotype and treatment. Images shown were chosen from a single representative experiment. The RT-qPCR analysis was performed on three biological replicates each with three technical replicates (*n* = 3). Statistical analysis of CM-H_2_DCFDA and GUS assay quantifications were carried out utilizing GraphPad Prism (GraphPad Software) performing *t*-test. Statistical analysis of RT-qPCR and stomatal closure levels were carried out utilizing GraphPad Prism with one-way ANOVA analysis followed by Šidák multiple comparison test. Statistical significance of differences was analyzed using *p* levels. *ns*, not significant; *p* levels > 0.05; *, **, ***, and **** *p* levels ≤ 0.05, 0.01, 0.001, and 0.0001, respectively.

## 3 Results

### 3.1 AtCuAOβ involvement in wound-induced stomatal closure

The constitutive as well as the MeJA-inducible expression of *AtCuAOβ* in guard cells (Ghuge et al., 2015c; Fraudentali et al., 2021b) may suggest a role of AtCuAOβ in the regulation of stomatal aperture levels under abiotic stress conditions. Considering this, the involvement of AtCuAOβ in the regulation of stomatal movement after both cotyledonary-leaf and root wounding was investigated using two different *Atcuaoβ* loss-of-function mutants, *Atcuaoβ.1* and *Atcuaoβ.3*.

**Figure 1:**
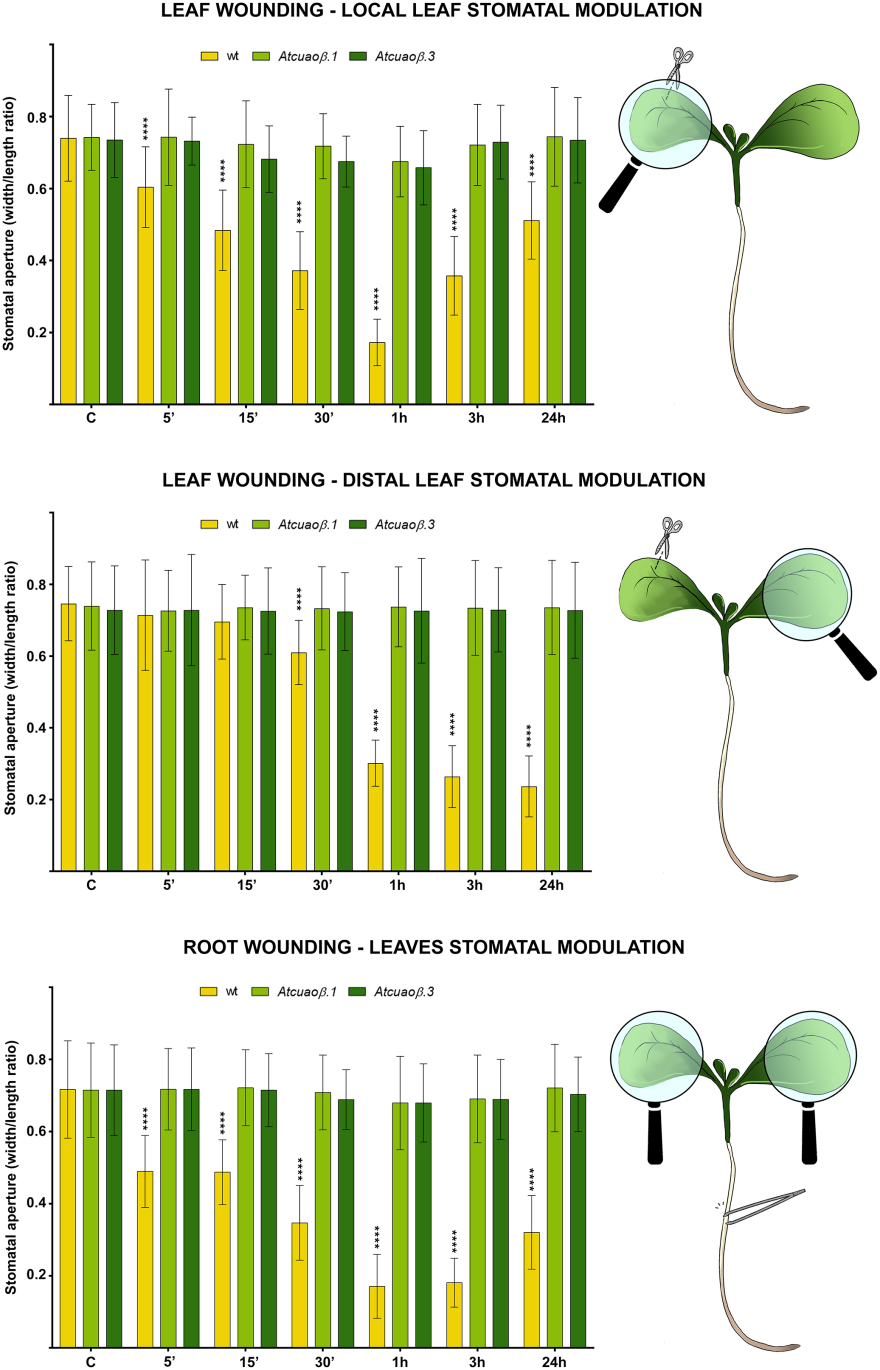
Effect of cotyledonary-leaf wounding and root wounding on stomatal pore modulation of 7-day-old WT and *Atcuaoβ.1* and *Atcuaoβ.3* mutant seedlings. Cotyledonary-leaf or root was injured and seedlings were incubated for 5 min, 15 min, 30 min, 1 h, 3 h, and 24 h. After cotyledonary-leaf wounding, analyses of stomatal pore modulation were carried out separately measuring stomata in wounded cotyledonary-leaves (local effect, top panel) and stomata in distal unwounded cotyledonary-leaves (distal effect, middle panel). After root wounding, analyses of stomatal pore modulation were carried out measuring stomata randomly chosen from both cotyledonary-leaves of each seedling (distal effect, bottom panel). Mean values ± SD (*n* = 15) are reported. Significance levels between WT unwounded seedlings (C) and WT wounded seedlings are reported. *p* levels have been calculated with one-way ANOVA analysis; *p* levels > 0.05; ****p levels ≤ 0.0001. If not shown, the statistical difference is not significant.

WT, *Atcuaoβ.1*, and *Atcuaoβ.3* 7-day-old seedlings were mechanically injured on the cotyledonary-leaf or the root and then stomatal aperture levels were analyzed after 5 min, 15 min, 30 min, 1 h, 3 h, and 24 h. Under control conditions, no significant differences in stomatal aperture levels were found between WT and *Atcuaoβ* seedlings. After cotyledonary-leaf wounding, stomata of WT seedlings in the wounded cotyledon started to close after 5 min and displayed a peak of closure of 75% after 1 h (**Figure 1**, top panel), while stomata in the unwounded cotyledon started to close after 30 min, displaying a peak of closure between 1 and 24 h (70%, as compared to WT unwounded seedlings) due to a distal wounding effect (**Figure 1**, middle panel). Instead, after cotyledonary-leaf wounding, *Atcuaoβ* mutants did not show any differences in stomatal aperture levels, neither in the wounded nor in the unwounded cotyledon, as compared to both WT and *Atcuaoβ* unwounded seedlings. Likewise, after root wounding, stomata of WT seedlings started to close after 5 min showing a peak of closure between 1 and 3 h (75%, as compared to WT unwounded seedlings), suggesting a systemic communication from root to leaf (**Figure 1**, bottom panel), while *Atcuaoβ* seedlings did not show any differences in stomatal aperture levels after root wounding, as compared to both WT and mutant unwounded seedlings.

**Figure 2:**
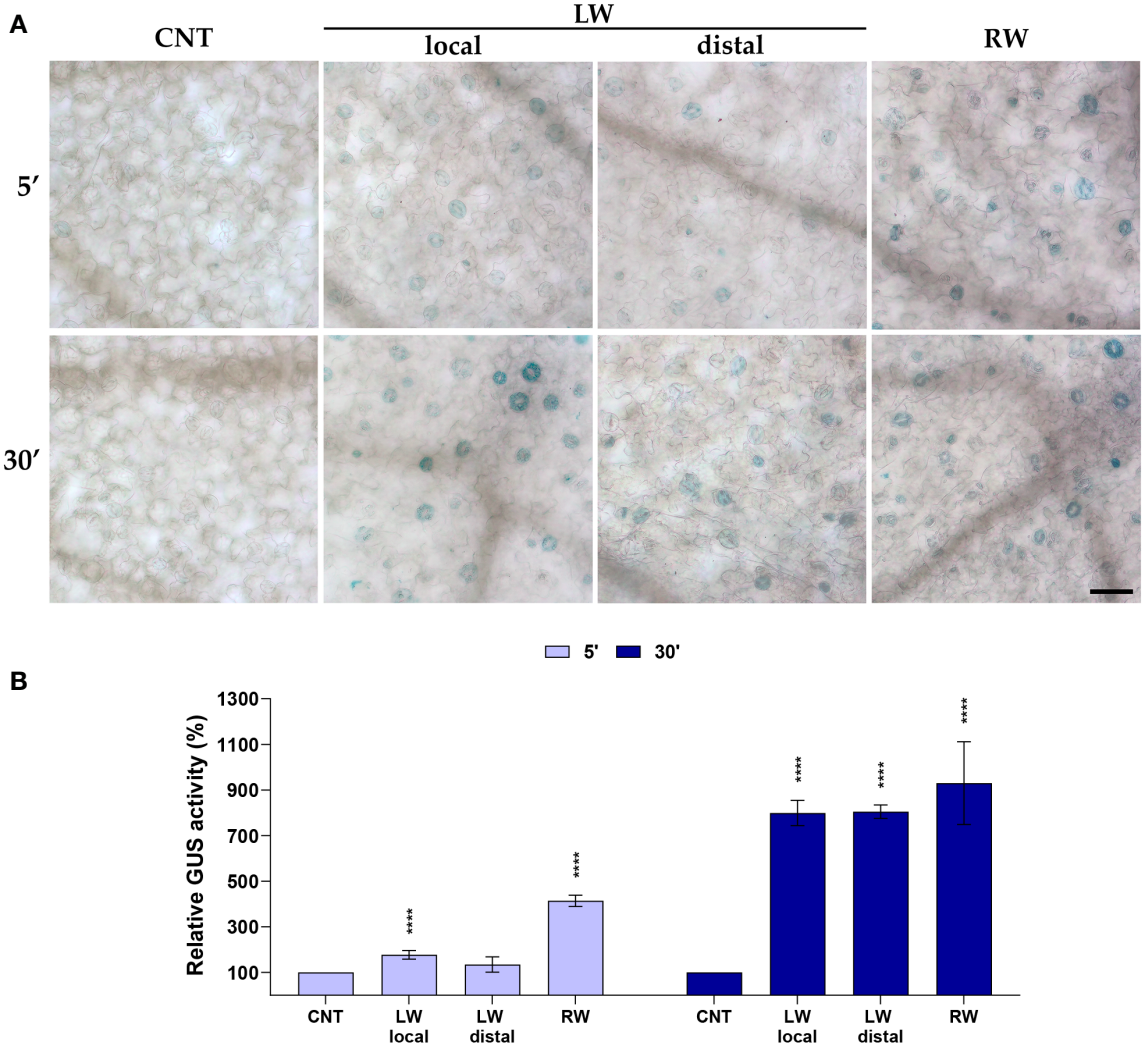
Analysis of *AtCuAOβ* tissue-specific expression pattern after cotyledonary-leaf or root wounding. Light microscopy analyses of GUS staining were carried out in 7-day-old *AtCuAOβ-promoter::GFP-GUS* transgenic seedlings. Stomata of control unwounded cotyledons (CNT), wounded cotyledons (LW local), distal unwounded cotyledons (LW distal) and cotyledons of root wounded seedlings (RW) were observed after 5 and 30 min from the onset of injuries. The staining reaction proceeded for 40 min. Micrographs are representative of those obtained from fifteen leaves from three independent experiments **(A)**. Quantification of changes in GUS activity in guard cells **(B)**. Mean values ± SD (*n* = 45) are reported. Significance levels between unwounded control seedlings (CNT) and wounded seedlings (LW, RW) are reported. p levels have been calculated with the t-test analysis; *p* levels > 0,05; **** *p* levels ≤ 0,0001. If not shown, the statistical difference is not significant. CNT, control; LW, leaf wounding; RW, root wounding. Bar = 50 μm.

To further investigate the involvement of AtCuAOβ in wound-induced stomatal closure, the effect of cotyledonary-leaf and root wounding on the *AtCuAOβ* expression profile was analyzed using *AtCuAOβ-promoter::GFP-GUS* transgenic seedlings through the histochemical assay of *β*-glucuronidase activity (GUS assay, **Figure 2**). Data were collected after 5 min and 30 min from the onset of injuries, which represent stomatal closure starting points induced by local leaf and root wounding (5 min) and distal leaf wounding (30 min), respectively, as shown in **Figure 1**. After leaf wounding, a weak GUS staining was detected in guard cells already 5 min after the injury, lower in the unwounded cotyledon than in the wounded one, while a strongly intensified GUS staining was clearly detected after 30 min in both wounded and unwounded cotyledons. After root wounding, a strong GUS staining was immediately detected already after 5 min and persisted strongly after 30 min. Unwounded seedlings showed a feeble promoter activity.

### 3.2 RBOHD involvement in wound-induced stomatal closure

The constitutive expression of *RBOHD* in guard cells together with its role as an apoplastic ROS source would suggest its involvement as well in generating a wound-associated signal that leads to stomatal closure. Considering this, the involvement of RBOHD in the regulation of stomatal movement after both cotyledonary-leaf and root wounding was investigated in *rbohd* loss-of-function mutant seedlings.

**Figure 3:**
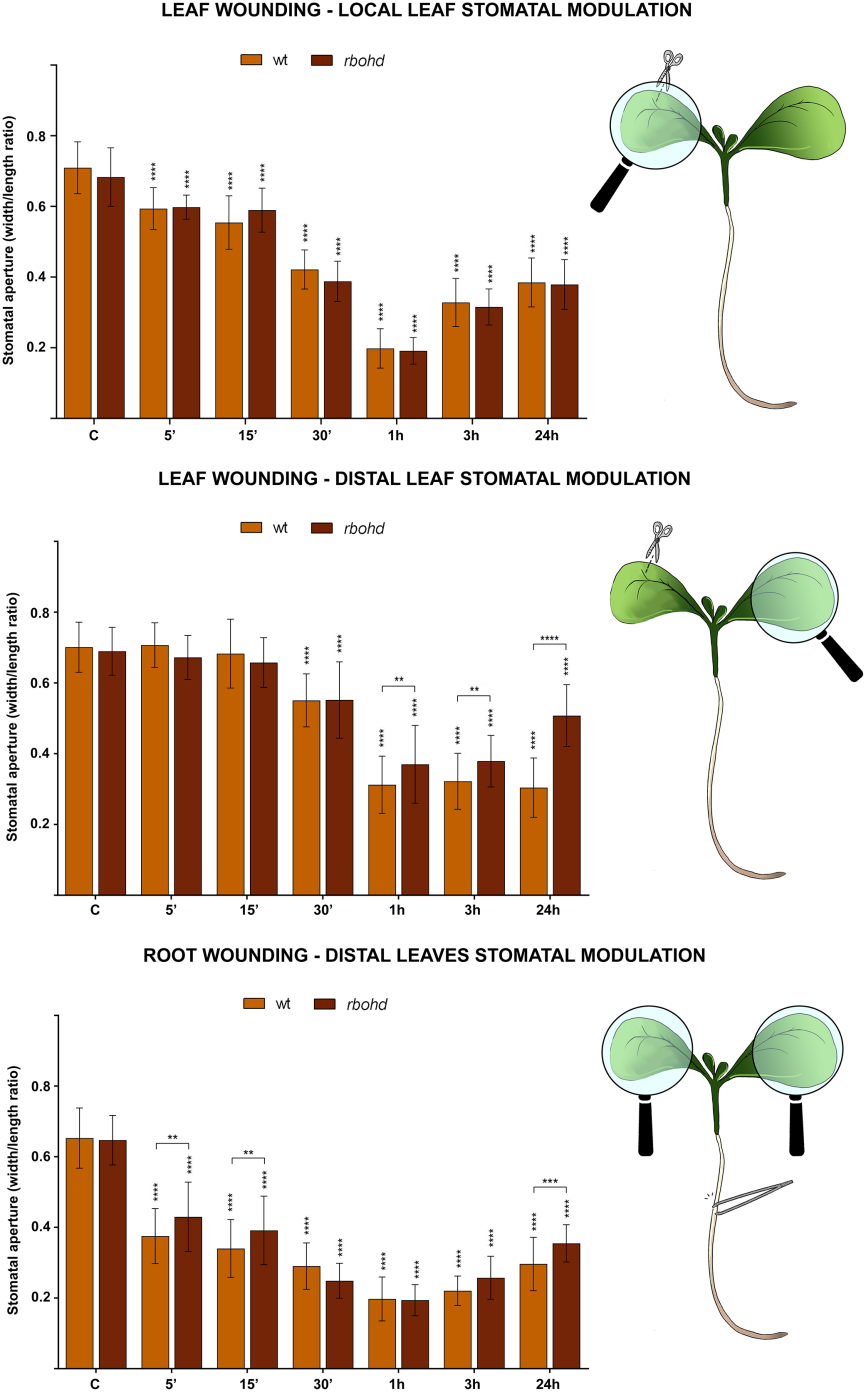
Effect of cotyledonary-leaf wounding and root wounding on stomatal pore modulation of 7-day-old WT and *rbohd* mutant seedlings. Cotyledonary-leaf or root was injured and seedlings were incubated for 5 min, 15 min, 30 min, 1 h, 3 h, and 24 h. After cotyledonary-leaf wounding, analyses of stomatal pore modulation were carried out separately measuring stomata in wounded cotyledonary-leaves (local effect, top panel) and stomata in distal unwounded cotyledonary-leaves (distal effect, middle panel). After root wounding, analyses of stomatal pore modulation were carried out measuring stomata randomly chosen from both cotyledonary-leaves of each seedling (distal effect, bottom panel). Mean values ± SD (*n* = 15) are reported. Significance levels between unwounded seedlings (C) and wounded seedlings of each genotype are reported. *p* levels have been calculated with one-way ANOVA analysis; *p* levels > 0.05; **, ***, and **** *p* levels ≤ 0.01, 0.001, and 0.0001, respectively. If not shown, the statistical difference is not significant.

WT and *rbohd* 7-day-old seedlings were mechanically injured on the cotyledonary-leaf or the root and then stomatal closure levels were analyzed after 5 min, 15 min, 30 min, 1 h, 3 h, and 24 h. Under control conditions, no significant differences in levels of stomatal aperture were found between WT and *rbohd* mutants. After leaf wounding, stomata of *rbohd* seedlings in the wounded cotyledon showed closure levels similar to WT seedlings at all time points considered, while stomata in the distal cotyledon started to close after 30 min but showed a peak of closure of only 50% after 1 h (in respect to the peak of 70% in WT) and started to reopen immediately after (**Figure 3**, top and middle panel). After root wounding, stomata of *rbohd* seedlings started to close after 5 min, although they were not as closed after 15 min as those of WT seedlings. Similar to WT, stomata of *rbohd* root wounded seedlings showed a peak of closure of 75% after 1 h but started to reopen earlier, as they were closed at a lower extent than WT stomata after 24 h (**Figure 3**, bottom panel).

To further investigate the specific role played by the D isoform compared to other RBOH isoforms in wound-triggered systemic stomatal modulation, the effect of wounding on stomatal movement in *rbohd* mutants was also evaluated in the presence of diphenyleneiodonium chloride (DPI), an inhibitor of all RBOH enzyme activities.

**Figure 4:**
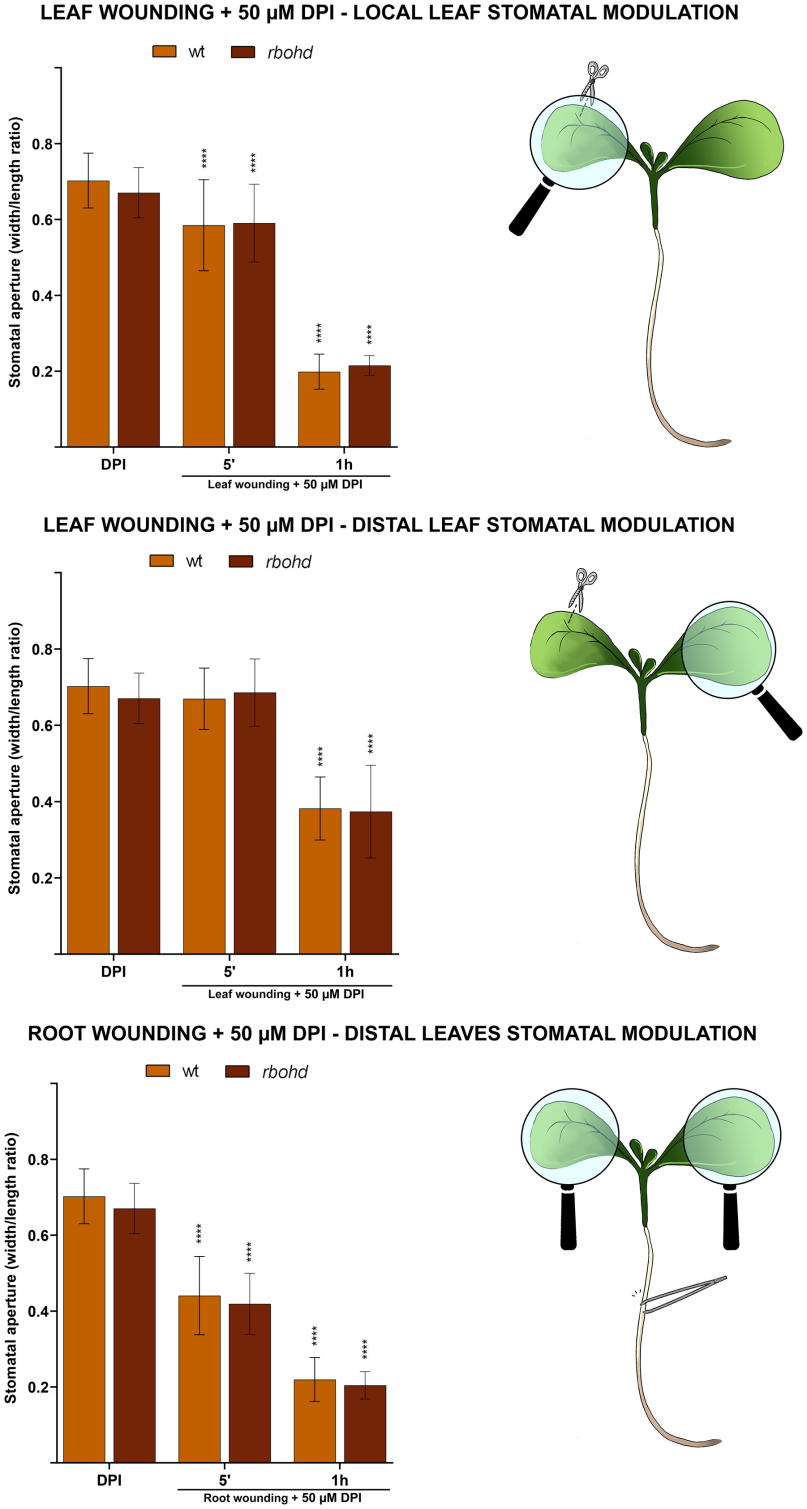
Effect of 50 μM DPI combined with cotyledonary-leaf wounding or root wounding on stomatal pore modulation of 7-day-old WT and *rbohd* seedlings. Cotyledonary-leaf or root was injured and seedlings were incubated in the presence of 50 μM DMTU for 5 min and 1 h. After cotyledonary-leaf wounding, analyses of stomatal pore modulation were carried out separately measuring stomata in wounded cotyledonary-leaves (local effect, top panel) and stomata in distal unwounded cotyledonary-leaves (distal effect, middle panel). After root wounding, analyses of stomatal pore modulation were carried out measuring stomata randomly chosen from both cotyledonary-leaves of each seedling (distal effect, bottom panel). Mean values ± SD (*n* = 15) are reported. Significance levels between unwounded and wounded DPI-treated seedlings of each genotype are reported. *p* levels have been calculated with one-way ANOVA analysis; *p* levels > 0.05; ****p levels ≤ 0.0001. If not shown, the statistical difference is not significant.

WT and *rbohd* 7-day-old seedlings were mechanically injured on the cotyledonary-leaf or the root and then incubated in the presence of 50 μM DPI for 5 min or 1 h (**Figure 4**). DPI alone did not significantly affect stomatal aperture levels both in unwounded WT and in mutant seedlings. Moreover, DPI also did not affect stomatal closure extent of injured *rbohd* seedlings, as no statistically relevant differences were found between DPI-treated and untreated wounded *rbohd* seedlings, neither locally nor systemically (**Figures 3** and **4**), suggesting the non-participation in wound-induced stomatal closure of other RBOH isoforms. However, DPI treatment partially reversed distal stomatal closure in WT seedlings, which show the same closure extent of *rbohd* wounded DPI-untreated seedlings (**Figures 3**, **4**; middle and bottom panel). Indeed, the effect of DPI was evident after 1 h in the leaf-to-leaf response and after 5 min in the root-to-leaf response (**Figure 4**), corresponding to time points of stomatal closure dynamics in which differences between WT and *rbohd* were found (**Figure 3**). These results are particularly relevant considering that *RBOHF*, but not *RBOHA*, other two leaf-localized RBOH isoforms, showed a significant compensatory induction of the constitutive expression level in unwounded *rbohd* seedlings (**Figure S1**), possibly functional in other physio-pathological events.

### 3.3 ROS level increase in guard cells mediated by AtCuAOβ and RBOHD is associated with wound-induced stomatal closure

The effect of wounding on stomatal movement in *Atcuaoβ* and *rbohd* mutants was also evaluated in the presence of the H_2_O_2_-scavenger DMTU to analyze the contribution of AtCuAOβ- and RBOHD-mediated ROS production.

**Figure 5:**
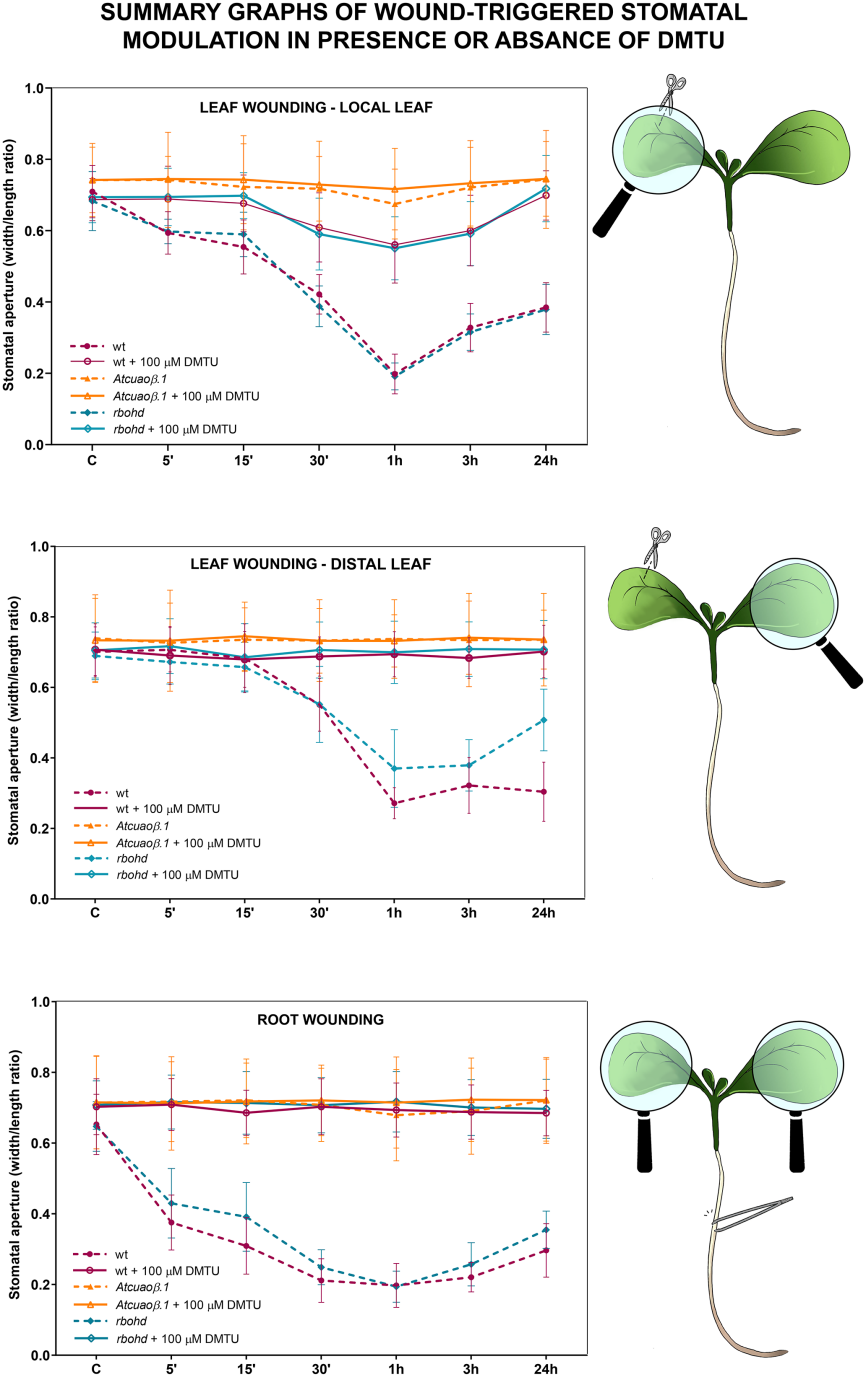
Summary graphs of data from **Figures 1**, **3**, and **S1**, showing stomatal aperture modulation by local or distal wounding and/or DMTU treatment in WT, *Atcuaoβ*, and *rbohd* mutants.

WT, *Atcuaoβ*, and *rbohd* 7-day-old seedlings were mechanically injured on the cotyledonary-leaf or the root and then incubated in the presence of 100 μM DMTU for 5 min, 15 min, 30 min, 1 h, 3 h, and 24 h (**Figure S2**; **Figure 5**). DMTU alone did not significantly affect stomatal aperture levels both in unwounded WT and in mutant seedlings. However, DMTU treatment reversed completely both root and distal leaf wound-induced stomatal closure in WT and *rbohd* seedlings, restoring the width/length ratio at the level of unwounded seedlings (**Figure S2** and **Figure 5**, middle and bottom panels), whereas it reversed almost completely local leaf wound-induced stomatal closure in WT and *rbohd* seedlings, restoring pore aperture levels at approximately 80% in respect to unwounded seedlings between 30 min and 3 h (**Figure S2** and **Figure 5**, top panel). As expected, DMTU treatment did not significantly affect stomatal aperture levels in *Atcuaoβ* seedlings neither after cotyledonary-leaf nor after root wounding.

**Figure 6:**
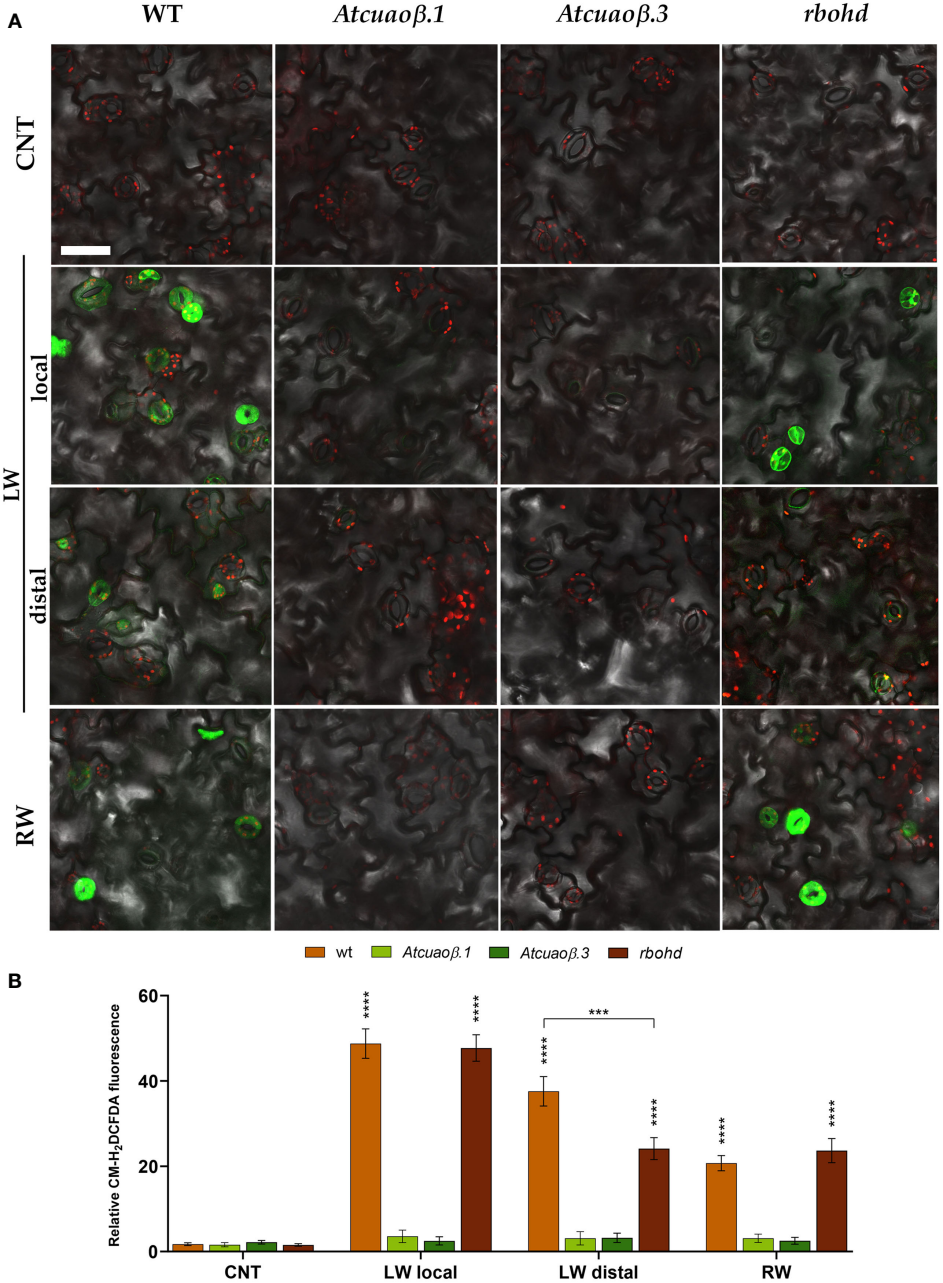
ROS levels in guard cells of 7-day-old seedlings after cotyledonary-leaf or root wounding. *In situ* ROS detection by LSCM analyses after CM-H_2_DCFDA staining was carried out in cotyledonary-leaves of WT, *Atcuaoβ.1*, *Atcuaoβ.3*, and *rbohd* seedlings. Stomata of unwounded cotyledons (CNT), wounded cotyledons (LW local), distal unwounded cotyledons (LW distal), and cotyledons from root wounded seedlings (RW) were observed after 1 h from the onset of injuries. Micrographs are representative of those obtained from five independent experiments, each time analyzing cotyledonary-leaves from five seedlings per genotype and treatment **(A)**. Quantification of relative fluorescence intensity of CM-H_2_DCFDA in guard cells **(B)**. Mean values ± SD (*n* = 15) are reported. Significance levels between unwounded control seedlings (CNT) and wounded seedlings (LW, RW) are reported. *p* levels have been calculated with the t-test analysis; *p* levels > 0,05; *** and **** *p* levels ≤ 0,001 and 0,0001, respectively. If not shown, the statistical difference is not significant. Bar = 50 µm. CNT, control; LW, leaf wounding; RW, root wounding.

To further investigate the contribution of AtCuAOβ and RBOHD in the modulation of stomatal closure through wound-induced ROS production, ROS levels in guard cells were visualized by LSCM using a chloromethyl derivative of 2’,7’-dichlorodihydrofluorescein diacetate (CM-H_2_DCFDA). WT, *Atcuaoβ*, and *rbohd* 7-day-old seedlings were mechanically injured on the cotyledonary-leaf or the root with (**Figure S3**) or without (**Figure 6**) 100 µM DMTU. Under control conditions, no fluorescence was detected in guard cells of WT, *Atcuaoβ*, and *rbohd* seedlings. Following cotyledonary-leaf or root wounding, a strong fluorescence was detected in stomata of WT seedlings, both locally and systemically, although after cotyledonary-leaf wounding, the signal was less intense in stomata of the unwounded cotyledon than in stomata of the wounded one. Interestingly, no fluorescence was detected in stomata of *Atcuaoβ* seedlings, either after cotyledonary-leaf or root wounding. Similar to WT, after cotyledonary-leaf wounding, a strong fluorescence was detected in wounded cotyledon stomata of *rbohd* seedlings, but a less intense signal was observed in unwounded cotyledon stomata. Moreover, a strong fluorescence was detected after root wounding in cotyledon stomata of *rbohd* seedlings (**Figure 6**). No fluorescence was detected in either condition combined with DMTU (**Figure S3**).

## 4 Discussion

Mechanical injury and abiotic and biotic stresses trigger a rapid systemic signal transduction process that activates different acclimation and defense mechanisms in distal tissues within minutes of stress sensing at the local tissues (Fichman et al., 2019; Kollist et al., 2019; Fichman and Mittler, 2020). Among systemic responses to wounding, root xylem remodeling (Fraudentali et al., 2018; Fraudentali et al., 2019) and stomatal closure (Devireddy et al., 2020) could represent strategies to enhance water uptake and counteract the excessive water loss caused by the injury. ROS, especially H_2_O_2_, are well-known second messengers of stress-induced Ca^2^-mediated signaling in guard cells (Pei et al., 2000; Kwak et al., 2003). Among apoplastic ROS generators, both RBOHD and AtCuAOβ have been shown to be involved in stress-mediated modulation of stomatal closure. Indeed, it has been reported that RBOHD is required in the signaling cascade leading to systemic, but not local, stomatal closure triggered by leaf wounding in 5-week-old Arabidopsis plants (Devireddy et al., 2020), while AtCuAOβ is shown to be involved in MeJA-mediated stomatal closure in 7-day-old Arabidopsis seedlings (Fraudentali et al., 2021b).

In this study, we present evidence of the prominent role of AtCuAOβ in local and systemic stomatal closure triggered by mechanical injury and that RBOHD has a role in systemic, but not local, modulation of stomatal closure triggered by mechanical injury. As revealed by the analysis of stomatal closure modulation, WT seedlings respond both locally and systemically to cotyledonary-leaf and root wounding (**Figures 1**, **3**, and **5**), while seedlings of *Atcuaoβ* mutants show a complete unresponsiveness in all these conditions, clearly evidencing that wound-induced local and systemic stomatal responses are both AtCuAOβ-dependent (**Figures 1**, **5**). Moreover, the partial unresponsiveness of *rbohd* seedlings in both leaf-to-leaf and root-to-leaf wound-triggered modulation of stomatal closure, along with their WT-like phenotype in response to local wounding (**Figures 3**, **5**), reveals the AtCuAOβ-dependent involvement of RBOHD in systemic but not local responses, which acts downstream of and cooperates with AtCuAOβ in the oxidative burst elicitation. The total unresponsiveness of *Atcuaoβ* mutants to local and distal injury demonstrates that stomatal closure is completely impaired in the absence of AtCuAOβ activity, which also hinders RBOHD participation in systemically induced stomatal closure. However, the only partial impairment of the systemic stomatal response in deficiency of RBOHD activity reveals its cooperation in reinforcing the oxidative burst downstream of AtCuAOβ, which is present in *rbohd* mutants and may act as a starter in the oxidative burst occurrence, in turn promoting subsequent RBOHD-mediated ROS production. Our data are supported by previous findings, showing the involvement of RBOHD in leaf-to-leaf but not local wound-triggered signaling (Devireddy et al., 2020), and firstly reveal its involvement in root-to-leaf wound-triggered signaling. Moreover, treatment of WT and *rbohd* seedlings with DPI, a well-known inhibitor of RBOH enzyme activities, inhibits stomatal closure in WT at a comparable extent than in *rbohd* mutants, whose response is otherwise not affected by the treatment, suggesting the non-participation of other RBOH isoforms, especially the leaf-localized RBOHF (Morales et al., 2016), and in line with the hypothesis that AtCuAOβ and RBOHD are the main players in the systemic wound-induced stomatal closure. This finding is particularly relevant considering that the expression of *RBOHF* shows a significant compensatory induction in unwounded *rbohd* seedlings (**Figure S1**) and that its cooperation with RBOHD in driving light stress-induced local and systemic ROS signaling has been reported (Zandalinas et al., 2020).

This work further demonstrates that the specific involvement of RBOHD and AtCuAOβ in local and systemic stomatal responses is due to their role as ROS generators. Indeed, data showed that in WT and *rbohd* injured seedlings, the H_2_O_2_ scavenger DMTU completely reverted the systemic stomatal closure, while it was not able to completely revert the local stomatal closure between 30 min and 3 h, which are the time points in which stomata result closed to a maximum extent (**Figure S2** and **Figure 5**). As expected, given the unresponsiveness of *Atcuaoβ* mutants to wounding, DMTU treatment did not affect their stomatal aperture levels in either condition (**Figure S2** and **Figure 5**). Possibly, the partial reversion of local wound-triggered stomatal closure in WT and *rbohd* seedlings could be explained by DMTU ineffectiveness in completely removing H_2_O_2_ produced in injured cotyledons when the stimulus induces the maximum level of closure, or alternatively, it could be due to the co-occurrence of H_2_O_2_-independent mechanisms involving other events correlated to CuAO activity, i.e., the aminoaldehyde production or changes in PA homeostasis. Again, the prominent role of AtCuAOβ-derived H_2_O_2_ in local and systemic modulation of stomatal closure triggered by wounding is further supported by the absence of ROS accumulation detected with CM-H_2_DCFDA in guard cells of *Atcuaoβ* injured seedlings in all conditions after 1 h from the onset of treatment (**Figure 6**, *Atcuaoβ.1/Atcuaoβ.3* LW local, LW distal, RW) that for systemic responses can be possibly explained by the lack of signal perception of the leaf-to-leaf and root-to-leaf wound-mediated communication. At the same time, the auxiliary role of RBOHD-derived ROS exclusively in systemic, but not local, stomatal closure modulation triggered by wounding is evidenced by the slight increase of ROS accumulation in guard cells of *rbohd* injured seedlings in distal cotyledons (**Figure 6**, *rbohd* LW distal), probably due to AtCuAOβ activity, and by the comparable levels of ROS accumulation in wounded cotyledons of WT and *rbohd* injured seedlings (**Figure 6**, WT/*rbohd* LW local). Moreover, considering the ROS accumulation in guard cells of *rbohd* seedlings injured in the root after 1 h from the onset of treatment, it is not unexpected to observe a strong accumulation of ROS (**Figure 6**, WT/*rbohd* RW) since, as shown in the analysis of stomatal closure levels, after 1 h, *rbohd* mutants close at the same extent of WT (**Figure 3**, bottom panel), possibly as a consequence of a very fast root-to-leaf signal propagation, starting already 5 min after injury perception in the root. On the contrary, the delayed temporal pattern of leaf-to-leaf signal propagation may result in a delayed RBOHD-driven ROS production as well, responsible for the weaker signal detected in distal cotyledons of *rbohd* injured seedlings at the same time point here considered.

**Figure 7:**
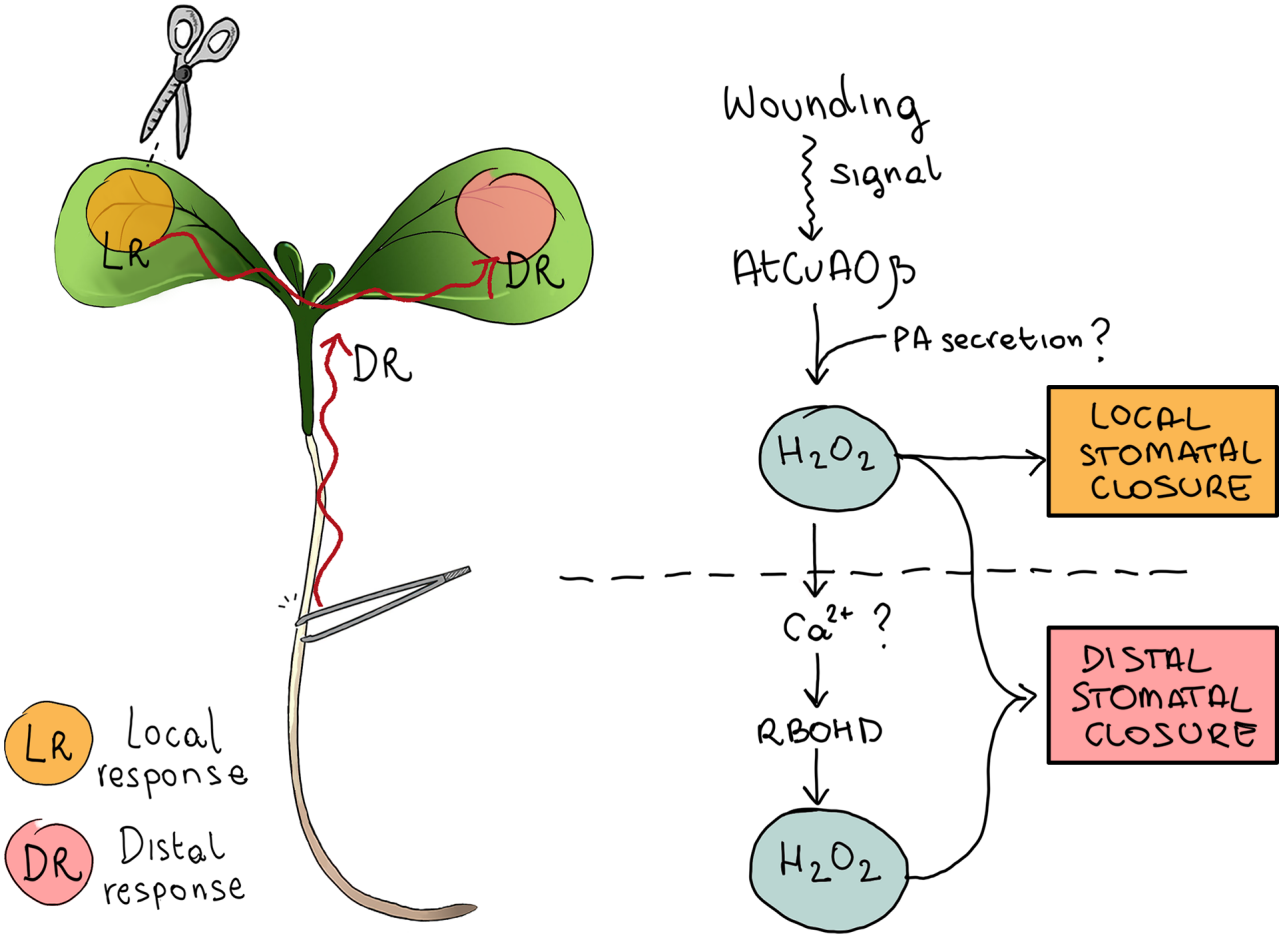
Schematic representation of the hypothetical signaling involved in wound-induced stomatal closure. AtCuAOβ-mediated PA oxidation in the cell wall of stomatal guard cells triggers local stomatal closure and represents a key step in the early phase of the oxidative burst responsible for systemic stomatal closure. Wound-induced PA secretion in the cell wall could be responsible for the rapid early induction of AtCuAOβ-driven oxidative burst. In distal responses, AtCuAOβ-driven H_2_O_2_ production activates ROS-dependent Ca^2+^ channels, thus increasing cytosolic Ca^2+^ levels, in turn inducing RBOHD-mediated ROS production, which is an essential component in the oxidative burst of systemic stomatal closure.

As a whole, these data lead us to hypothesize that AtCuAOβ and RBOHD may establish a feed-forward ROS systemic amplification loop, which culminates in the execution of distal mechanical damage-induced stomatal closure in Arabidopsis seedlings. In this hypothetical scenario, AtCuAOβ positively influences the activity of RBOHD, and RBOHD is downstream of AtCuAOβ in the relay of events that controls ROS accumulation in wound-induced stomatal closure (**Figure 7**). The coordination between NADPH oxidases and AOs has already been conceptualized previously. Indeed, AO and RBOH enzymes are involved in many physiological processes, raising the possibility that they are functionally interlinked in the control of ROS homeostasis (Benkő et al., 2022). In this regard, Gémes et al. hypothesized that NADPH oxidases and apoplastic PAOs do not have parallel pathways for ROS production, but they do form a cross talk in regulating ROS homeostasis (Gémes et al., 2016). The convergent action of AOs and RBOHs may also occur in the control of stomatal aperture levels under stress conditions, as stress factors induce stomatal regulation *via* ROS production. Moreover, the involvement of the specific RBOHD and AtCuAOβ isoforms in long-distance responses has also been previously reported. Specifically, Miller et al. (2009) showed that RBOHD is essential for rapid systemic signaling in response to wounding, heat, cold, intense light, or increased salinity and that its involvement is necessary for the establishment of the cell-to-cell ROS wave (Mittler et al., 2011). Likewise, our previous work provides evidence that the AtCuAOβ-driven H_2_O_2_ production mediates the early root protoxylem differentiation signaled by shoot-to-root long-distance communication upon leaf wounding (Fraudentali et al., 2020a). These lines of evidence, together with data herein reported, suggest a role of AtCuAOβ in water balance homeostasis by modulating coordinated adjustments in anatomical and functional features of xylem tissue and guard cell aperture levels in damaged seedlings, which could be related to a rapid improvement in seedling functionality in water uptake during stress conditions. In this context, the existence and the exact nature of the hypothesized cascade of events that starts from local injury perception and leads to distal stress response, propagating along plant tissues and organs and involving the AtCuAOβ–RBOHD relationship in ROS production, still need further investigation, as it may lead to a better understanding of how plants can rapidly fine-tune their proper functioning, acclimation, or survival depending on the environmental context.

## Data availability statement

The original contributions presented in the study are included in the article/**Supplementary Material**. Further inquiries can be directed to the corresponding author.

## Author contributions

AC, IF and CP conceived experiments. IF and CP performed experiments. AC, IF and CP wrote the manuscript. IF, CP, RD, PT, RA and AC reviewed and edited the manuscript. RA, PT and AC acquired funding. All authors contributed to the article and approved the submitted version.
