# Supplementary material for: Distinct role of AtCuAOβ- and RBOHD-driven H2O2 production in wound-induced local and systemic leaf-to-leaf and root-to-leaf stomatal closure
Source: Front Plant Sci. 2023 Apr 21;14:1154431. doi: 10.3389/fpls.2023.1154431 (PMC10160378; doi:10.3389/fpls.2023.1154431)
Supplement: Supplementary file 1 [file DataSheet_1.pdf]

Supplementary Material

Distinct role of AtCuAOβ- and RBOHD-driven H_2O_2 production in wound-induced local and systemic leaf-to-leaf and root-to-leaf stomatal closure

Ilaria Fraudentali, Chiara Pedalino, Riccardo D'Incà, Paraskevi Tavladoraki, Riccardo Angelini, Alessandra Cona*

*** Correspondence:**
alessandra.cona@uniroma3.it

1 Supplementary Figures

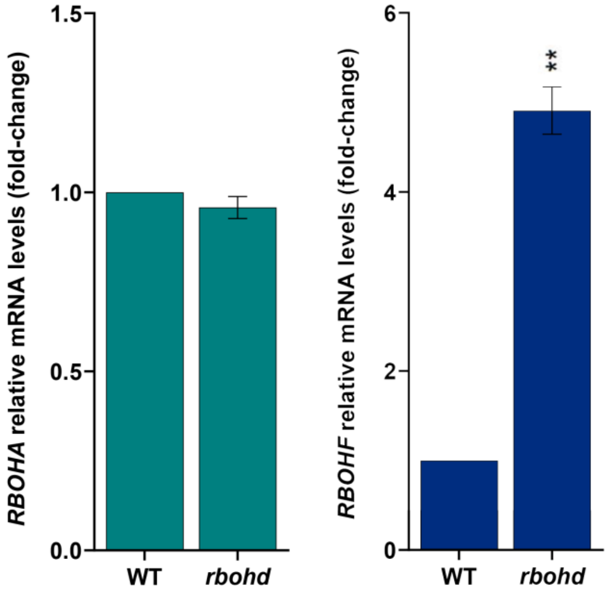

Supplementary Figure 1. Analysis of *RBOHA* and *RBOHF* constitutive expression by RT-qPCR in 7-day-old WT and *rbohd* seedlings. The reported values of fold-inductions in *rbohd* are relative to WT, with the value for WT assumed to be one. Data are the result of three biological replicates, each with three technical replicates (mean values ± SD; $n = 3$). The significance levels between relative mRNA levels are reported only when $p \leq 0.05$. **, p levels ≤ 0.01.

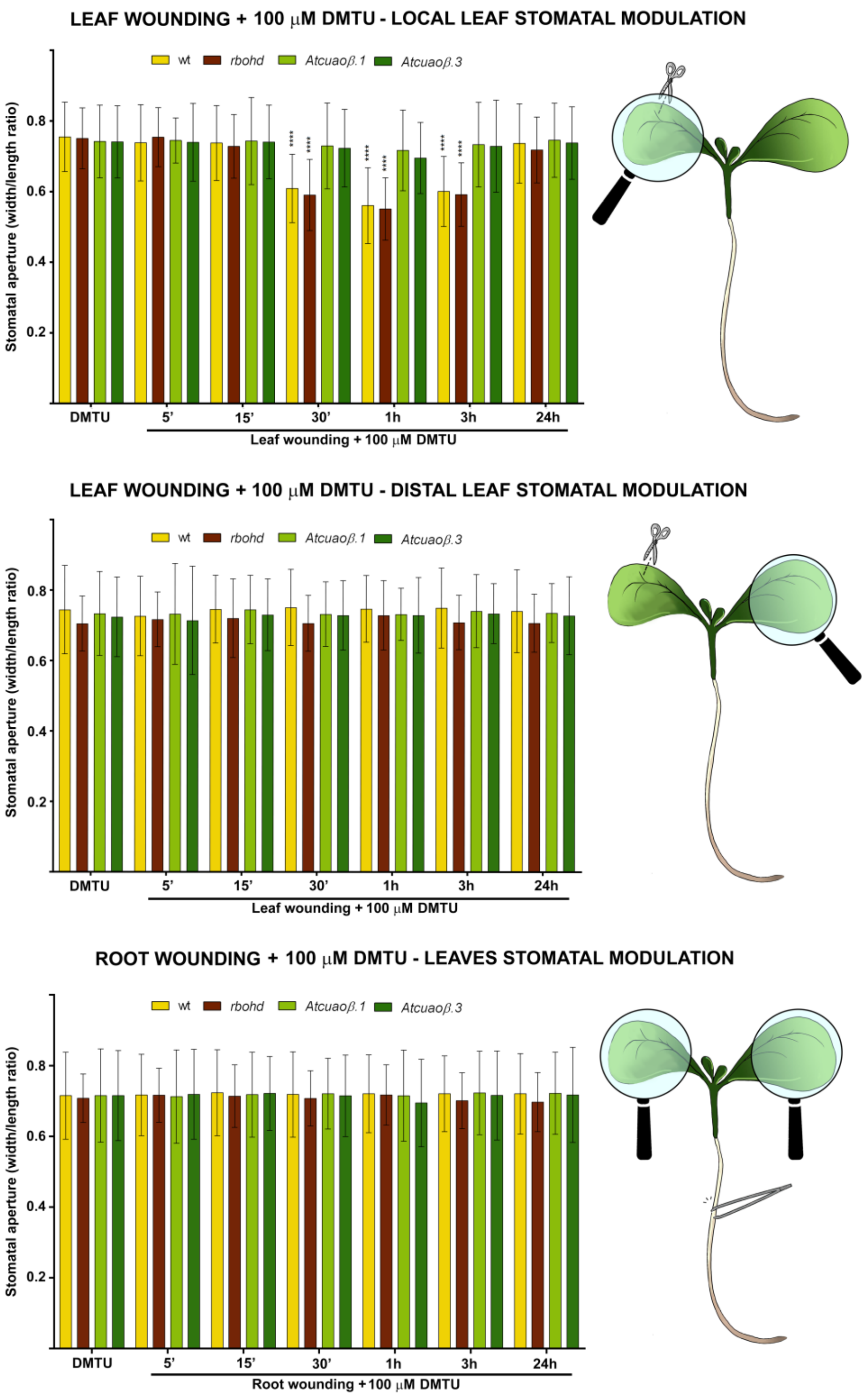

Supplementary Figure 2. Effect of 100 μM DMTU combined with cotyledonary-leaf wounding or root wounding on stomatal pore modulation of 7-day-old WT, *Atcuaoβ*, and *rbohd* mutant seedlings. Cotyledonary-leaf or root was injured and seedlings were incubated in presence of 100 μM DMTU for 5, 15, 30 min, 1, 3 and 24 h. After cotyledonary-leaf wounding, analyses of stomatal pore modulation

were carried out separately measuring stomata in wounded cotyledonary-leaves (local effect, top panel) and stomata in distal unwounded cotyledonary-leaves (distal effect, middle panel). After root wounding, analyses of stomatal pore modulation were carried out measuring stomata randomly chosen from both cotyledonary-leaves of each seedling (distal effect, bottom panel). Mean values ± SD (n = 15) are reported. Significance levels between unwounded and wounded DMTU treated seedlings of each genotype are reported. p levels have been calculated with one-way ANOVA analysis; p levels > 0,05; **** p levels ≤ 0,0001. If not shown, the statistical difference is not significant.

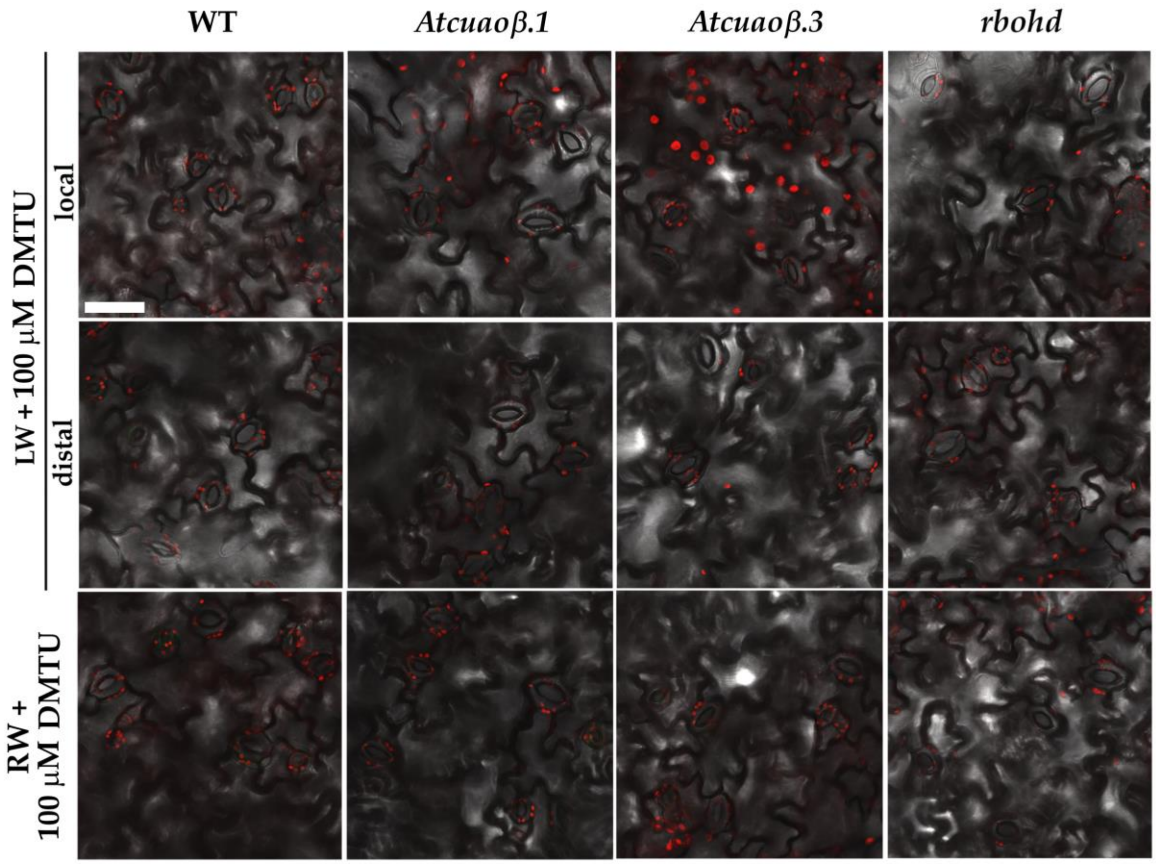

Supplementary Figure 3. Effect of 100 μM DMTU combined with cotyledonary-leaf wounding or root wounding on ROS production in guard cells of 7-day-old seedling. *In situ* ROS detection by LSCM analyses after CM-H_2DCFDA staining were carried out in cotyledonary-leaves of WT, *Atcuaoβ.1*, *Atcuaoβ.3*, and *rbohd* seedlings. Stomata of wounded cotyledons (LW local), distal unwounded cotyledons (LW distal) and cotyledons from root wounded seedlings (RW) were observed 1 h after the injury combined with DMTU treatment. Micrographs are representative of those obtained from five independent experiments, each time analyzing cotyledonary leaves from five seedlings per genotype and treatment. Bar = 50 µm. DMTU, N,N^1-dimethylthiourea; LW, leaf wounding; RW, root wounding.
